# Mutation in *CEP63* co-segregating with developmental dyslexia in a Swedish family

**DOI:** 10.1007/s00439-015-1602-1

**Published:** 2015-09-23

**Authors:** Elisabet Einarsdottir, Idor Svensson, Fahimeh Darki, Myriam Peyrard-Janvid, Jessica M. Lindvall, Adam Ameur, Christer Jacobsson, Torkel Klingberg, Juha Kere, Hans Matsson

**Affiliations:** Department of Biosciences and Nutrition, and Center for Innovative Medicine, Karolinska Institutet, Huddinge, Sweden; Department of Psychology, Linneaus University, Växjö, Sweden; Department of Neuroscience, Karolinska Institutet, Solna, Sweden; Bioinformatics Infrastructure for Life Sciences (BILS), Stockholm University, Stockholm, Sweden; Science for Life Laboratory, Stockholm University, Stockholm, Sweden; Department of Biochemistry and Biophysics, Stockholm University, Stockholm, Sweden; Uppsala Genome Center, Uppsala University, Uppsala, Sweden; Department of Pedagogy, Linneaus University, Växjö, Sweden; Molecular Neurology Research Program, Research Programs Unit, University of Helsinki, Helsinki, Finland; Folkhälsan Institute of Genetics, Helsinki, Finland

## Abstract

Developmental dyslexia is the most common learning disorder in children. Problems in reading and writing are likely due to a complex interaction of genetic and environmental factors, resulting in reduced power of studies of the genetic factors underlying developmental dyslexia. Our approach in the current study was to perform exome sequencing of affected and unaffected individuals within an extended pedigree with a familial form of developmental dyslexia. We identified a two-base mutation, causing a p.R229L amino acid substitution in the centrosomal protein 63 kDa (*CEP63*), co-segregating with developmental dyslexia in this pedigree. This mutation is novel, and predicted to be highly damaging for the function of the protein. 3D modelling suggested a distinct conformational change caused by the mutation. CEP63 is localised to the centrosome in eukaryotic cells and is required for maintaining normal centriole duplication and control of cell cycle progression. We found that a common polymorphism in the *CEP63* gene had a significant association with brain white matter volume. The brain regions were partly overlapping with the previously reported region influenced by polymorphisms in the dyslexia susceptibility genes *DYX1C1* and *KIAA0319*. We hypothesise that CEP63 is particularly important for brain development and might control the proliferation and migration of cells when those two events need to be highly coordinated.

**Electronic supplementary material:**

The online version of this article (doi:10.1007/s00439-015-1602-1) contains supplementary material, which is available to authorized users.

## Introduction

Disabilities in cognitive processes such as dyslexia are a major problem worldwide, although estimates of their prevalence vary greatly (Katusic et al. 2001; Roeleveld et al. 1997). Developmental dyslexia (DD) is the most common learning problem in children, manifesting as deficiencies in reading and writing skills (Francks et al. 2002; Kere 2014; Shaywitz et al. 1990). These difficulties arise despite normal intelligence, presence of educational opportunities and independently of the social environment. Individuals affected by DD show impairments in multiple correlated cognitive processes, especially the relation between letters and sounds, but the difficulties can often be traced to phonological processes (Elliott and Grigorenko 2014; Tunmer and Greaney 2010) which typically remain stable over time (Svensson and Jacobson 2006). Secondary consequences may include problems in reading comprehension and reduced interest in reading. DD can occur in multiple generations in families and studies have shown a relative high heritability (Shaywitz 1998).

DD is considered a complex trait in which variants in several genes may influence brain development. Some of these genes may execute their effect by interacting with environmental factors (Schumacher et al. 2007). In such cases, the power of genetic assays to identify the causative genetic factors is relatively low. This means that genetic studies of DD must either be very extensive or strategic to maximise the findings of the molecular biological analyses. Previous research has implicated genetic factors contributing to increased risk (Kere 2014) of developing reading and writing deficiencies although some studies using unrelated individuals have not been replicated. The difficulty in finding strong genetic association between common polymorphisms and DD was recently illustrated by the findings of the EU-funded project NeuroDys. This consortium used individual genotyping of 19 single-nucleotide polymorphisms (SNPs) in *DYX1C1*, *DCDC2*, *KIAA0319*, and the *MRPL19*/*C2ORF3* locus using case–control samples from multiple research groups from throughout Europe. Despite this ambitious and multi-center approach, no SNPs reached genome-wide significance (Becker et al. 2014) and no single SNPs were associated with DD in samples from more than one population. This may potentially be explained by differences in diagnosis between countries, genetic heterogeneity among the different populations or insufficient power due to the heterogeneity of the disease, or a combination of all those factors.

Family studies have proven to be valuable in identifying genetic factors associated with increased risk in common traits (Brunham and Hayden 2013), including dyslexia (Hannula-Jouppi et al. 2005; Taipale et al. 2003). In fact, many of the genes originally identified in families inheriting dyslexia have led to new insights in the function of the genes (Lamminmaki et al. 2012; Tammimies et al. 2013) and multiple studies have replicated the original findings (Scerri and Schulte-Korne 2010). In the current study, we describe our approach to identify a novel potential susceptibility gene for DD. This is based on exome sequencing of selected individuals from an extended family from Sweden with familial DD. Due to the diagnosis and family structure, we assumed an autosomal dominant inheritance of DD with high or complete penetrance. Our aim was to identify rare, high-risk disease variants that would, in this particular pedigree, strongly influence disease risk. Identification of such variants might give us new leads on genes and pathways that are important also for the more common forms of dyslexia. Here, we describe and characterise a novel two-base mutation in *CEP63* resulting in an amino acid substitution co-segregating with DD suggesting *CEP63* as a new candidate gene for DD. Correlation of genotype to phenotype using computational structure predictions can aid the identification of potential damaging or deleterious non-synonymous variants (Kumar and Purohit 2012). As protein structures for CEP proteins are typically not available, we utilised in silico tools for 3D modelling of the predicted wild-type and mutated CEP63 proteins. Finally, we discuss the potential functional implications of the mutation and suggest future prospects to evaluate the effect of the mutated CEP63 on cellular functions.

## Materials and methods

### Ethics statement

Ethical permission for genetic analysis and collection of test data in this study was granted by the Faculty of Medicine Research Ethics Committee at the University of Gothenburg (L-225-99) and the regional ethics committee (EPN) Stockholm (Dnr 2013/214-31/3). Informed consent was given by all participating individuals.

### Participants

**Fig. 1:**
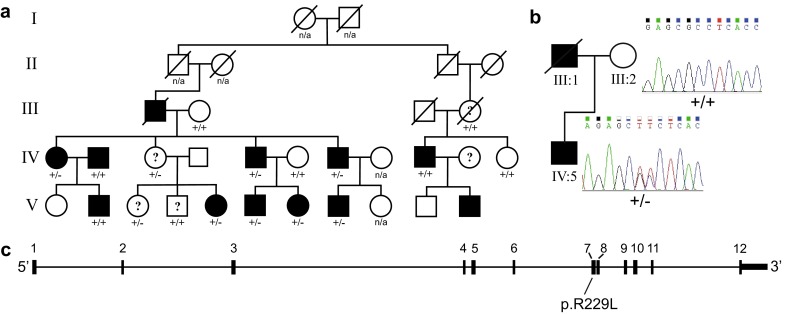
Exome sequencing in a family with DD shows affected individuals co-segregating a novel *CEP63* mutation. **a** Family members with confirmed DD are denoted by *black filled symbols*. *White symbols* indicate individuals for which testing excluded DD diagnosis. *Question marks* indicate uncertain DD diagnosis. Generations are marked with* roman numbers* to the* left* of the* image* and individuals are *counted from left to right*. Affected individuals IV:1, IV:5, IV:7, IV:9, V:6, V:7 and V:8, as well as healthy individuals III:2, IV:6 and IV:11 were selected for exome sequencing. Family members for whom DNA was available with a Sanger sequencing-verified c.686_687insdelTT *CEP63* mutation are shown with ±, representing heterozygous mutation, whereas +/+ represents wild-type *CEP63* alleles. No testing for DD was available for individuals marked with *n/a*. **b** Sanger sequencing chromatograms for representative wild-type (+/+) and c.686_687delinsTT (±) family members. **c** Schematic representation of the *CEP63* gene region. Exons are *numbered* and depicted as *filled black boxes*. The c.686_687delinsTT mutation predicts a p.R229L in exon 7

A three-generation family (*n* = 62) was recruited from a local reading and writing center in southern Sweden as described previously (Svensson et al. 2011). The proband was tested and diagnosed with severe reading and writing disabilities. Thirty-five percent of the family members (22/62 individuals) were diagnosed with dyslexia (Svensson et al. 2011), twelve of them are represented in Fig. 1a. A drawing of the full pedigree is available in Fig. S1.

### Criteria for developmental dyslexia and assessment procedure

Particular weight was given to phonological assessments. Likewise, a self-reported history of reading difficulties was important concerning the affected status. To be regarded as dyslexic, the participants had to fail at least two tests of phonological ability plus a positive history of reading problems. In addition, if there was no self-report of reading and writing disabilities, the participant had to fail at least three phonological tests and two manifest tests of literacy such as word recognition and orthographic choice. A test performance below 1 standard deviation (SD) compared to the norms on these tests was considered a fail, as it has been the case in several previous studies (Nopola-Hemmi et al. 2001; Schulte-Korne et al. 1998; van der Leij et al. 2001). The interview and the questionnaire were also used as a complement to either confirming or rejecting a diagnostic of dyslexia, especially in uncertain cases and for children below 13 years.

Fifty-six of the participating family members completed tests in the assessment battery. For 10- to 13-year olds, the tests were adapted to their age. Two 9-year-old children, who were interviewed and completed some reading tests, were regarded as dyslexic. For one child below the age of 9, only personal records from the parents were available. Children below the age of 7 were not included in the investigation.

In total, 10 reading and spelling tests (word recognition, oral word reading, letter chain, orthographic choice, phonological choice, pseudo-word reading, spoonerism, spelling, reading comprehension, listening comprehension) covering both phonological and orthographic decoding skills, self-reported history of reading ability, years in school and short-term memory were included. Furthermore, data from a non-verbal intelligence test (Raven et al. 1983) were available to exclude low extremes (below the 10th percentile) on this test even though no participants were excluded for that reason. The test battery in the present investigation was similar to that used in many other genetic studies concerning dyslexia (Fagerheim et al. 1999; Fisher et al. 1999; Grigorenko et al. 1997; Kaplan et al. 2002; Olson et al. 1989). The participants that failed on two to four tests were classified as uncertain cases if there were no previous reports of reading and writing disabilities.

### DNA samples and ion proton exome sequencing

Blood samples were initially collected from the participants and DNA was prepared as previously described (Svensson et al. 2011). We re-measured available DNA concentration of available genomic DNA from family members using Qubit BR dsDNA assay (Life Technologies, USA). Individuals III:2, IV:1, IV:5, IV:6, IV:7, IV:9, IV:11, V:6, V7 and V:8 were selected for exome sequencing (Fig. 1a). All DNA samples used for exome sequencing were tested for DNA degradation using Genomic DNA ScreenTape analysis (Agilent Technologies, USA). Subsequently, 100 ng non-degraded genomic DNA per sample was used in AmpliSeq library preparations followed by Ion Proton (Life Technologies, USA) sequencing at the Science for Life Laboratory, Uppsala, Sweden, according to standard protocols. AmpliSeq target regions are available upon request. The Ion Proton sequencing was performed on P1 chips producing 200-bp sequence reads.

### Variant calling, validation and analysis

Sequence reads were aligned to the hg19 genome assembly using a proprietary Ion Proton pipeline (Life Technologies, USA). Single-nucleotide variants (SNVs) and small insertions and deletions (indels) were called for each sample individually using the Torrent Suite Software (Life Technologies, USA). We aimed to identify highly penetrant variants contributing to a monogenic trait in the family members. First, we selected variants found in all affected family members after exome sequencing. Next, to enrich for novel and very rare variants, we filtered out any SNVs present in an in-house database (Ameur et al. 2014) of 700 exome data variants (Uppsala Genome Center, Uppsala, Sweden). SNV validation was carried out using Sanger capillary sequencing using standard protocols (KIGene, Karolinska Institutet, Solna, Sweden). Based on the inheritance pattern of DD in the family, the transmission of SNVs among affected individuals was analysed assuming a dominant inheritance pattern with high penetrance.

### Analysis of CEP63 conservation and expression

The UniProtKB/Swiss-Prot database (www.uniprot.org/uniprot/) was queried for the amino acid sequences of CEP63 homologues in multiple species to understand the conservation and likely impact of the p.R229L amino acid change. The amino acid sequences for human CEP63 homologues in orangutan, crab-eating macaque, rat, mouse, chicken and zebrafish were aligned for comparison.

RNA expression of *CEP63* was determined by CAGE in phase 1 and 2 of FANTOM5 data, adapted from the Zenbu promoterome browser at fantom.gsc.riken.jp/zenbu/. The human protein atlas (http://www.proteinatlas.org/) was used to assess tissue protein expression profiles.

### Pathway analysis

We performed a search for CEP63 interaction partners using the Ingenuity Pathway Analysis (IPA) software (Qiagen, CA, USA). Network enrichment analysis was performed using FunCoup (Functional Coupling tool, http://funcoup.sbc.su.se/search/) for all interactions partners found in the IPA analysis (Schmitt et al. 2014).

### Structural modelling of CEP63

To investigate the probable effects of the p.R229L mutation in CEP63, we analysed the predicted 3D structure of the protein either with the Arginine (R) or the Leucine (L) at position 229 of the protein. We utilised the RaptorX (Kallberg et al. 2012) tool (from the University of Chicago, US, raptorx.uchicago.edu) for 3D structure predictions with default parameters. This method aims at optimising the modelling to the specific target being used, while integrating, into the modelling, a variety of biological signals. Multiple secondary structure templates are also used, if possible, to aid in the modelling of the specific target sequence.

Pdb files from RaptorX were visualised further in Jmol: an open-source Java viewer for chemical structures in 3D (http://www.jmol.org/).

### Brain imaging

We utilised brain imaging in a set of 76 healthy controls to test for correlation of *CEP63* genetic variants to brain structure, in particular white matter volume. These individuals have been studied previously (Darki et al. 2012, 2014) and were genotyped using whole-genome SNP arrays (Affymetrix Genome-wide SNP array 6.0). To select variants for the brain imaging analysis, we chose all genotyped SNPs within the *CEP63* gene region and used Haploview version 4.2 (Barrett et al. 2005) and the tagger option with default setting (pair-wise marker correlation *r*^2^ > 0.8). Minimum minor allele frequency was set to 20 %. Two SNPs, rs7619451 and rs9868985, together tagged (*D*′ = 1) the linkage disequilibrium (LD) region within *CEP63*.

Structural brain images were collected by three-dimensional T1-weighted magnetization prepared rapid gradient echo (MP-RAGE) sequence with TR = 2300 ms, TE = 2.92 ms, field of view of 256 × 256 mm^2^, 256 × 256 matrix size, 176 sagittal slices, and 1 mm^3^ isotropic voxel size. Scanning was repeated three times with the same parameters, each 2 years apart. To segment the brain into grey matter, white matter and CSF, Diffeomorphic Anatomical Registration Through Exponentiated Lie Algebra (DARTEL) was performed. The white matter segmented images were then smoothed with an 8-mm Gaussian kernel and were analysed using a flexible factorial design using the Statistical Parametric Mapping (SPM) software package. Each SNP was entered as main factor and the model was corrected for age, sex, handedness and total white matter volume. The gene interactions by age and sex were also considered in the model.

## Results

### Targeted exome sequencing and validation

High molecular weight genomic DNA from seven affected and three unaffected individuals was used in exome sequencing (Fig. 1a). The sequencing produced an average of 37 (29-39) million reads for all samples resulting in an average sequence coverage of 90.3 % (88.4–92.4 %) on target regions and a mean sequence depth of 73–130× (average 99×) over each base. Aligned sequencing reads were submitted to the European Nucleotide Archive (ENA) at EMBL-EBI and are available as study accession number PRJEB9294.

The variant calling predicted on average 50556 SNPs per sample (SD = 6524). We hypothesised that the dominant inheritance pattern of DD in the pedigree is due to a heterozygous DNA mutation segregating with DD in all affected individuals. An exception from the dominant inheritance hypothesis was made for individual IV:9 (Fig. 1a). No phenotypic data was available from his mother and DD has a frequency of 5–10 % in the general population, it was thus unclear from which parent the DNA variant would be transmitted (Fig. 1a). Initial variant calling and filtration were performed on the first sequencing run of eight exomes using DNA from individuals IV:1, IV:5, IV:6, IV:7, IV:9, V:6, V:7 and V:8. We found in total 283 variants, of which 142 are non-synonymous, present in each and every affected individual (including IV:9) while not found in the unaffected (Table S1). Thereafter, we applied a series of variant filtering steps to pinpoint the variant segregating with DD in the family. To enrich for rare or novel variants any of the 283 variants present in an in-house database of 700 exomes were removed. The remaining 16 variants all had a minor allele frequency below 1 % (1000Gphase3EUR and ESP6500), as summarised in Table S2. To further restrict the number of variants shared exclusively among affected family members, a second exome sequencing run including unaffected individuals III:2 and IV:11 was performed. We chose to proceed with the analysis of variants in genes with experimentally validated brain expression profiles. These analyses resulted in a total of eight variants located in the *CEP63*, *DNAJC11*, *GET4*, *INTS5*, *PAQR9*, *RNF152, TNRC18* and *ZNF507* genes, five of which are non-synonymous.

Due to the inheritance pattern of DD in the pedigree, we chose to validate the five non-synonymous variants in the *CEP63*, *GET4*, *INTS5*, *RNF152* and *ZNF507* genes using Sanger sequencing and all individuals from the family (Fig. 1a) for which DNA was available. The evidence for all polymorphisms was supported by at least 20 sequence reads carrying the alternative allele (Table S2). The novel c.T775G (p.259C>G) variant in *GET4* was found to be monomorphic and thus represents an error in the exome sequencing or variant calling. A novel variant in *INT5S* (c.3026C>T, p.P1009L) was predicted to be present in individual IV:9 (Table S2). However, upon Sanger sequencing, the mother (III:4, uncertain affection status) was shown not to carry the variant and as the father is unaffected, the transmission of the variant from a common ancestor was not confirmed. Furthermore, unaffected individuals III:2 and IV:11 carried the predicted variant in *ZNF507* (c.634C>G p.P212A), leading us to exclude this polymorphism from further analysis (Table S2). The predicted variant in *RNF152* (c.359G>A, p.R120H) was also found in unaffected individual IV:6 and was thus also excluded from further analysis (Table S2).

A single non-synonymous variant was confirmed by Sanger sequencing to segregate with DD in the family using all available DNA samples. This mutation is a novel complex polymorphism involving two adjacent bases at position chr3:134264558-9 (build 37), resulting in a p.R229L c.686_687delinsTT (NM_001042383) variant in exon 7 (Fig. 1a–c) of the centrosomal protein 63 kDa (*CEP63*). The G/T variant predicting the non-synonymous amino acid substitution is very rare with a minor (T) allele frequency of 0.01 % in the European population (ExAC, http://exac.broadinstitute.org/). We found that that the chr3:134264558 position is highly conserved [Gerp (Cooper et al. 2005) score 5.53] and the G/T variant is predicted to be probably damaging by PolyPhen-2 (score 1) (Adzhubei et al. 2010) and by SIFT (score 0) (Kumar et al. 2009), see Table S2. Due to the amino acid substitution and potential functional significance of the c.686_687delinsTT in *CEP63*, we undertook further analysis of the CEP63 protein and the novel mutation.

### Differential expression profiles of CEP63 transcript variants

We investigated publicly available data on the RNA expression of *CEP63* to get an understanding of how dysregulation of CEP63 might contribute to risk of DD. The FANTOM5 promoterome data browser reveals that *CEP63* is mainly transcribed from a cluster of transcription start sites (TSSes) at the first exon of the full-length *CEP63* transcript. The expression of transcripts originating from this TSS cluster is best described as ubiquitous (Fig. S2). Another set of transcripts is transcribed from a region within the gene, resulting in transcripts starting from exons 4–9. These transcripts show a very different expression pattern, being mainly expressed in brain, particularly in the medial temporal gyrus and the substantia nigra (Fig. S3).

### Conservation and structural modelling of CEP63

**Fig. 2:**
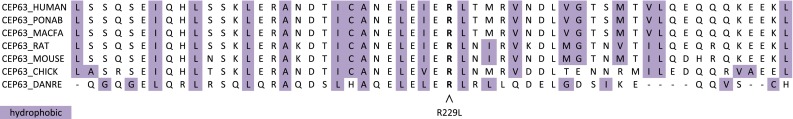
ClustalW alignment of the sequence of amino acids 200-255 of human CEP63 isoform 1 (Q96MT8-1) as well as the homologous sequence for orangutan (PONAB, *Pongo abelii*), crab-eating macaque (MACFA, *Macaca fascicularis*), rat (RAT, *Rattus norvegicus*), mouse (MOUSE, *Mus musculus*), chicken (CHICK, *Gallus gallus*) and zebrafish (DANRE, *Danio rerio*). Hydrophobic residues are marked in *purple*. The p.R229L mutation found to be co-segregating with DD in the current study is in *bold* and* marked* with an *arrow*

The c.686_687delinsTT mutation in *CEP63* results in an in-frame amino acid substitution (p.R229L). The position is highly conserved in vertebrates (Fig. 2). A sequence of regularly spaced hydrophobic residues in the proximity of pR229L seems to be conserved in all the species we studied, and changing the charged side chain of arginine to the hydrophobic side chain of a leucine could very plausibly interfere with this pattern.

**Fig. 3:**
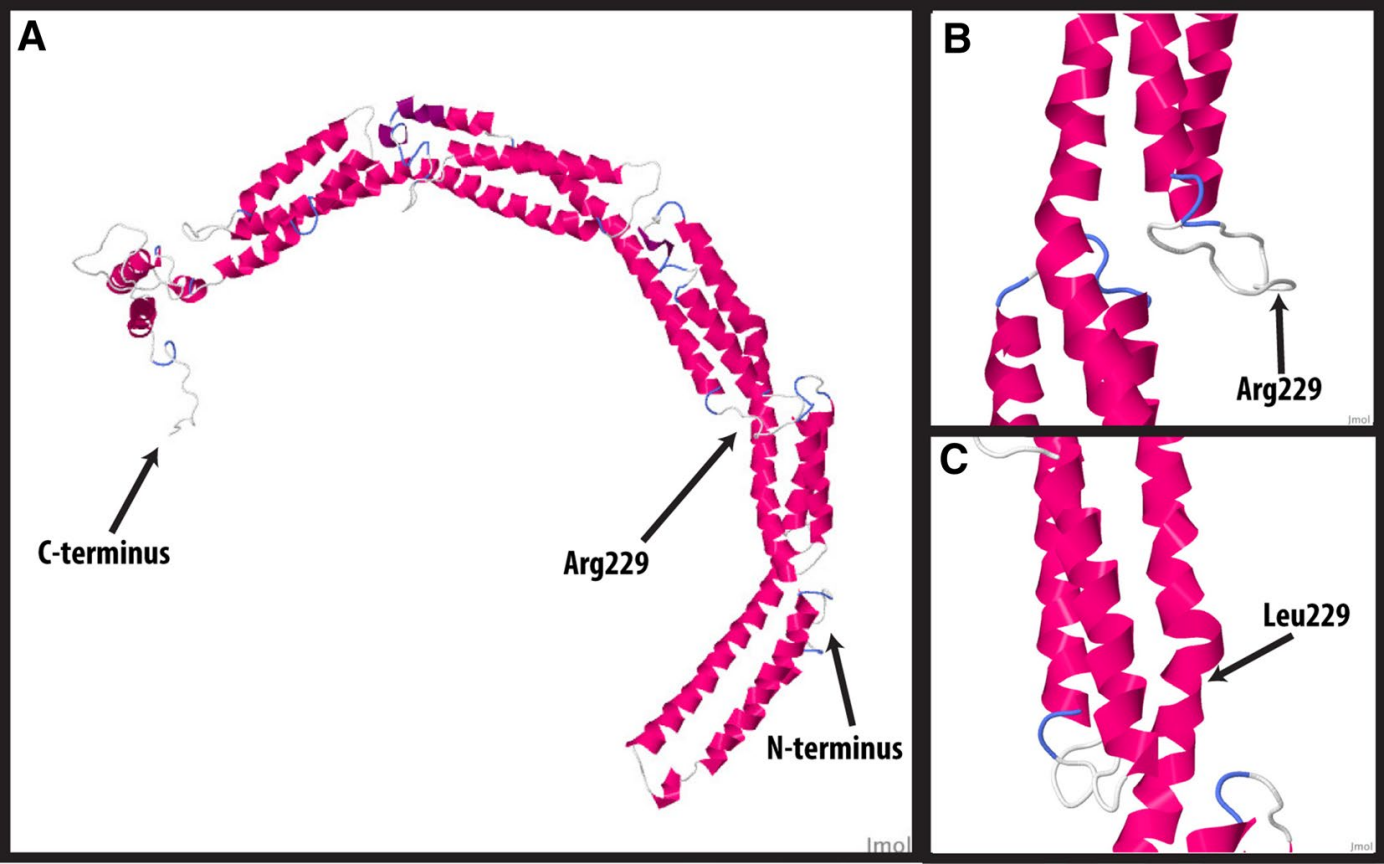
Predicted tertiary structure of wild-type (wt) CEP63 carrying an arginine at site 229 of the protein and mutated (mut) CEP63 with a leucine at the same amino acid position. In the wild-type protein (Arg229), amino acid 229 is part of a hinge segment between two coiled-coil domains, (**a**, **b**). In the mutated protein (**c**) (Leu229), this hinge segment is predicted to be lost. The prediction was made with the RaptorX tool (University of Chicago, US, http://www.raptorx.uchicago.edu) using the default parameters

To predict the structural defects in the tertiary structure of the mutated protein, we attempted to model the mutation in 3D. Our hypothesis was that the 3D structure of the protein might change slightly (conformation change, domain change, access of binding sites, etc.). In the prediction from this modelling, we could show that the mutated residue abolishes a hinge region between two coiled-coil regions (Fig. 3) thus disrupting the three-dimensional structure of CEP63. Pdb files of the results are available upon request.

### Pathway analysis and interaction partners of CEP63

We used the Ingenuity Pathway Analysis (IPA) software for analyses of protein–protein interactions involving CEP63. Here, we present evidence of direct, experimentally validated, protein–protein interactions (i.e. no intermediate proteins) of CEP63 with 13 proteins: CEP152, DISC1, DST, DTNB, EXOC4, MACF1, NCOR2, NDEL1, PPP1R13B, PPP4R1, SMARCE1, SYNE1 and TBC1D15 (Table S3; Fig. S4). Despite extracting these interactions and applying a functional coupling analysis, using FunCoup, we did not reveal any direct interconnection among any of the 13 binding targets of CEP63. Based on these results, we speculate that each of the interacting partners might interact at separate cellular locations and/or time-point, with CEP63.

### Genetic effects on white matter volume

Developmental dyslexia is likely a neurological disease, and given the high brain expression of certain *CEP63* transcripts in human (Figs. S2, S3), we aimed at investigating the impact of genetic variations within *CEP63* on brain structure, more specifically on white matter volume. Here, we used available genotypes from a whole-genome SNP array and MRI data to correlate common variants with white matter volume in a set of 76 healthy controls (Darki et al. 2012, 2014). Interestingly, we showed that the AA/AC genotypes of rs7619451 are significantly associated with higher white matter volume in the right hemisphere overlapping with right superior longitudinal fasciculus and posterior part of corpus callosum (peak coordinate: 28, −55, 29; *p* = 0.0076, corrected at the cluster level with *p* < 0.01). The cluster of significant effect on white matter volume for rs7619451 in the right temporo-parietal region partly overlaps with a region previously found significant for rs3743204 and rs6935076 from the dyslexia candidate genes *DYX1C1*and *KIAA0319,* respectively (Fig. S5) (Darki et al. 2012). We extracted the mean white matter volume from the significant region in the right hemisphere as well as its homologous area in the left hemisphere. Rs7619451 was significantly associated with white matter volume in both left (*p* = 0.006) and right (*p* = 0.003) regions of interest.

Moreover, the association of rs7619451 with reading comprehension scores from those 76 individuals was assessed using a mixed linear model considering three longitudinal measures of reading ability, each collected two years apart. The reading score was entered as dependent variable, and the SNP genotype was set as a factor. Age, sex, handedness and the SNP interaction with age were considered as covariates. The main effect of rs7619451 [*F*_(1, 88.98)_ = 5.938, *p* = 0.017] as well as its interaction by age [*F*_(1,91.62)_ = 5.518, *p* = 0.021] was found significant for reading comprehension.

## Discussion

A previous whole-genome linkage analysis using SNP genotyping (10 k Affymetrix array) of the family members under study-yielded inconclusive results (Svensson et al. 2011). Here, we used phenotypic data and validated variants detected after exome sequencing of genomic DNA from key family members to identify the risk variant co-segregating with DD in this family. We could identify a mutation segregating with DD in *CEP63*, a gene not implicated in DD previously. The mutation found in the family is a two base-pair variant predicting a p.R229L substitution in CEP63 at a position between two of the six coiled-coil domains. CEP63 is, together with at least 30 other proteins of the CEP family, localised to the centrosome (Kumar et al. 2013), an organelle that functions as the major microtubule-organising center in eukaryotic cells and is essential for proper cell cycle progression. Several alternatively spliced variants of *CEP63* with a variable number of protein coding exons have been found. However, their biological validity has not yet been determined experimentally. Exon 7, which contains the mutation described here, is shared among a majority of the annotated *CEP63* transcripts. CEP63 is likely needed in all or most cells of the human body as seen from its ubiquitous expression. However, our observations of the TSSs of the various transcripts indicate that there may also be a tissue-specific set of transcripts that are uniquely important for the brain. If these transcripts are affected by the p.R299L variant, this may help explain why carriers of the mutation show developmental dyslexia but no other noticeable phenotype. Presently, we can only speculate about the exact mechanism for this effect.

Several studies report on a link between DD candidate genes and centrosome and cilia functions. *DYX1C1* was identified as a DD candidate gene more than a decade ago (Taipale et al. 2003) and has since then been replicated as a DD gene by genetic association studies. Interestingly, the murine homologue *Dyx1c1* is upregulated in ciliogenesis and exogenous DYX1C1 is localised to the centrosome (Hoh et al. 2012). Furthermore, we and others have linked reduced DYX1C1 levels to ciliary defects using a mouse model (Tarkar et al. 2013) and a zebrafish model system (Chandrasekar et al. 2013). Recent reports have described mutations in the dyslexia candidate gene *DCDC2* in individuals with renal-hepatic ciliopathy or recessive deafness (Grati et al. 2015; Schueler et al. 2015). We speculate that amino acid substitutions in centrosomal proteins and proteins involved in ciliogenesis can increase risk of developmental dyslexia. The amino acid position and the severity of the alterations in chemical properties of the mutated protein might govern the observed/resulting phenotypic effect.

As CEP63 is expressed in brain and involved in mechanisms of cell division, we hypothesised that individuals heterozygous for CEP63 mutations may present mild defects in neuronal cell migration and proliferation leading to the cognitive problems associated with DD. Defects in neuronal cell proliferation and migration can manifest as altered density of white matter in the brain. As demonstrated earlier, SNPs in DD candidate genes affect white matter volume in a cohort of normally developing children and young adults from Sweden (Darki et al. 2012). In this study, we saw that there was a significant correlation between genotypes of rs7619451, located in *CEP63*, and white matter volume in the right hemisphere of healthy individuals. The cluster of significant effect further overlapped with a region previously found to be significant for SNPs within the DD candidate genes *DYX1C1* and *KIAA0319* (Darki et al. 2012). While this does not directly prove the p.R229L mutation in CEP63 as the cause of DD in the family under study, it supports our hypothesis that subtle genetic variation in *CEP63* might contribute to normal variation in the mechanisms underlying DD. It also highlights further the emerging role of centrosome function in neuronal growth and maintenance.

Our Ingenuity Pathway Analysis shows that CEP63 directly interacts with multiple target proteins in the cell and these interactions are experimentally validated. The interacting targets could be subject to further analyses as it is tempting to speculate that the CEP63 mutation may alter the protein structure and result in reduced or loss of protein complex formation with one or several CEP63 binding proteins. Interestingly, the CEP63 p.R229L mutation is located within the CEP152 binding region (Firat-Karalar et al. 2014) and the mutation may possibly alter the protein–protein interaction between CEP63 and CEP152. This must, however, be verified experimentally.

The *CEP63* and *DEUP1* genes were produced from a single gene locus by gene duplication leading to proteins with related but specialised functions. The CEP63 paralog DEUP1 promotes mother centriole-independent centriole amplification in multi-ciliated differentiated cells (Zhao et al. 2013). Unlike DEUP1, CEP63 is required for normal mother centriole-dependent centriole duplication by recruitment of CEP152 and regulates mitotic entry through binding and recruitment of CDK1 to the centrosome (Brown et al. 2013; Loffler et al. 2011). Studies in U2OS cells revealed that CEP63 is localised at the proximal end of the paternal centriole where it forms a ring structure in complex with CEP57 and CEP152 (Lukinavicius et al. 2013). Interestingly, homozygosity for a nonsense mutation in *CEP63* was reported in three cousins, from a consanguineous Pakistani family, presenting with microcephaly and short stature (Seckel syndrome-6, OMIM 614728) (Sir et al. 2011). Microscopy imaging using fluorescent antibodies in primary human white blood cells showed partly disordered or incomplete CEP152 ring structures in CEP63^+/−^ individuals (carriers), or complete loss of CEP152 rings in CEP63^−/−^ cells (Sir et al. 2011). Recently, it was shown that mice deficient in Cep63 display a Seckel syndrome phenotype with reduced adult weight accompanied by mitotic errors leading to p53-dependent neuronal progenitor cell death (Marjanovic et al. 2015).

Also, it remains to be seen if any of the previously reported DD candidate gene products form protein complexes with centrosomal proteins in general and CEP63 in particular. Future in vitro assays using exogenous mutated CEP63 could be initiated to investigate relevant cellular processes, such as centriole duplication and neuronal differentiation. These experiments could reveal the effect of gene mutations on centrosome function and, potentially link mutations in *CEP63* to the phenotype of developmental dyslexia.

To conclude, we describe a novel *CEP63* mutation in an extended three-generation family from Sweden and suggest *CEP63* as a new candidate gene for DD. Further functional analysis of the mutated CEP63 protein may reveal aberrant cellular functions and implicate the centrosome as an important cellular structure in the biology underlying developmental dyslexia.

## Electronic supplementary material

Below is the link to the electronic supplementary material.

Supplementary material 1: Fig. S1 Pedigree of a six-generation family segregating developmental dyslexia. Family members with confirmed DD are denoted by black filled symbols. White symbols indicate individuals for which testing excluded DD diagnosis. Question marks indicate uncertain DD diagnosis. (PDF 695 kb)

Supplementary material 2: Fig. S2 FANTOM5 expression of ubiquitous transcription start sites (TSSes). The top panel shows the genomic context within *CEP63* (gene region marked in green) and the known transcripts below. The middle panel shows the levels of expression starting at each region. The green bars show the level of expression of *CEP63*. The cluster of TSSes at the start of *CEP63* is highlighted (grey), and the bottom panel shows where the transcripts from this specific TSS cluster are expressed (green bars, sorted by expression levels). The highest expression is in neutrophils (234.97 rle normalised tags/library), but expression is seen in most tested tissues (for clarity, only some of the tissues are shown). (TIFF 1608 kb)

Supplementary material 3: Fig. S3 FANTOM5 expression of neuronal TSS. The top panel shows the genomic context within which *CEP63* (gene region marked in green) lies and the known transcripts below. The middle panel shows the levels of expression starting at each region. The green bars show expression of *CEP63*. The cluster of TSSes inside the *CEP63* gene is highlighted (grey), and the bottom panel shows where the transcripts from this TSS cluster are expressed (green bars, sorted by expression levels). The highest expression is seen in the medial temporal gyrus (220.52 rle normalised tags/library) and only a few tissues show any expression > 10 rle normalised tags/library (an arbitrary cut-off for determining expression in a tissue). (TIFF 3256 kb)

Supplementary material 4: Fig. S4 Thirteen direct protein–protein binding partners for CEP63. All molecules were experimentally validated according to analysis criteria in the IPA software. The shapes of the individual proteins in the figure denote the different protein families that the binding partners belong to (e.g. protein phosphatases PPP4R1 and PPP1R13B). (PDF 864 kb)

Supplementary material 5: Fig. S5 Clusters (in yellow) from MRI data showing significant correlation between genotypes in rs7619451 and white matter volume in human brain. The AA/AC genotypes were significantly associated with larger white matter volume. The regions in the right hemisphere overlap with right superior longitudinal fasciculus and posterior part of corpus callosum (peak coordinate: 28, -55, 29; p = 0.0076, corrected at the cluster level with p < 0.01). (TIFF 1734 kb)

Supplementary material 6: Table S1 Table of predicted variants shared among affected individuals using exome sequencing. (XLSX 35 kb)

Supplementary material 7: Table S2 Table of shared predicted non-synonymous and synonymous variants after in-house database filtering. (XLSX 13 kb)

Supplementary material 8: Table S3 List of experimentally validated direct interacting protein partners to CEP63 based on Ingenuity analysis. (PDF 101 kb)
