# Supplementary figures and images for: Mutation in CEP63 co-segregating with developmental dyslexia in a Swedish family
Source: Hum Genet. 2015 Sep 23;134:1239–48. doi: 10.1007/s00439-015-1602-1 (PMC4628622; doi:10.1007/s00439-015-1602-1)

I

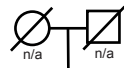

II

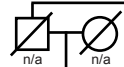

III

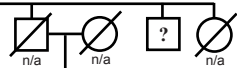

IV

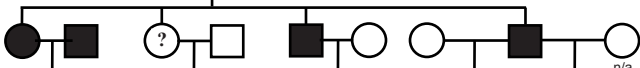

V

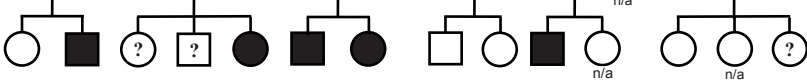

VI

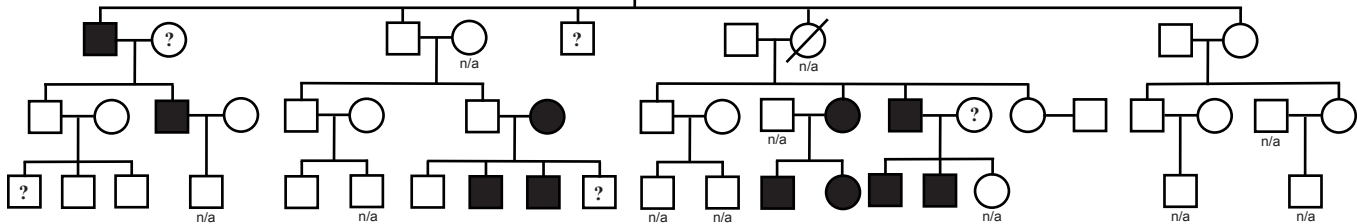

Supplement: Supplementary file 1 — Supplementary material 1: Fig. S1 Pedigree of a six-generation family segregating developmental dyslexia. Family members with confirmed DD are denoted by black filled symbols. White symbols indicate individuals for which testing excluded DD diagnosis. Question marks indicate uncertain DD diagnosis. (PDF 695 kb) [file 439_2015_1602_MOESM1_ESM.pdf]

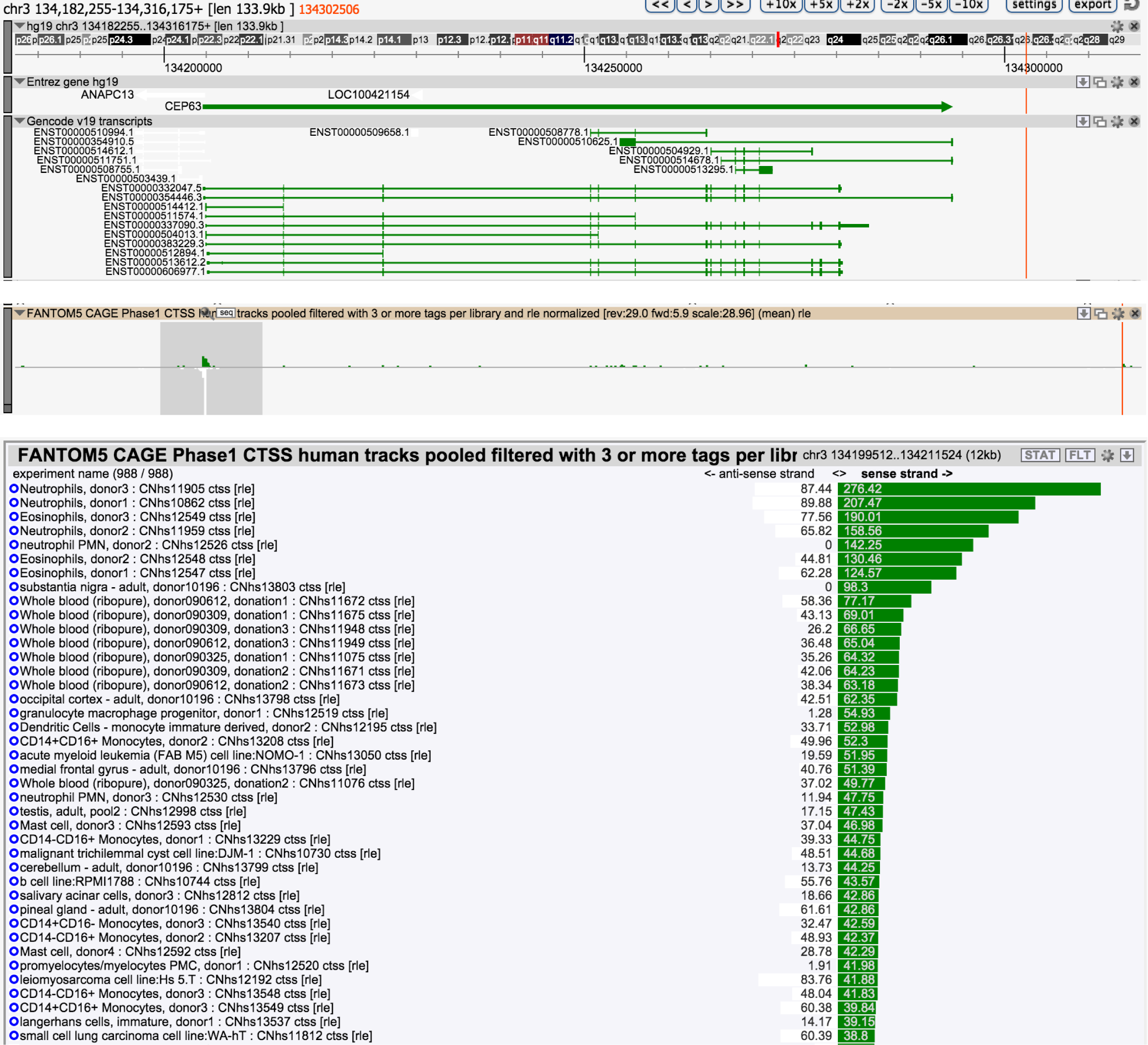

Supplement: Supplementary file 2 — Supplementary material 2: Fig. S2 FANTOM5 expression of ubiquitous transcription start sites (TSSes). The top panel shows the genomic context within CEP63 (gene region marked in green) and the known transcripts below. The middle panel shows the levels of expression starting at each region. The green bars show the level of expression of CEP63. The cluster of TSSes at the start of CEP63 is highlighted (grey), and the bottom panel shows where the transcripts from this specific TSS cluster are expressed (green bars, sorted by expression levels). The highest expression is in neutrophils (234.97 rle normalised tags/library), but expression is seen in most tested tissues (for clarity, only some of the tissues are shown). (TIFF 1608 kb) [file 439_2015_1602_MOESM2_ESM.tiff]

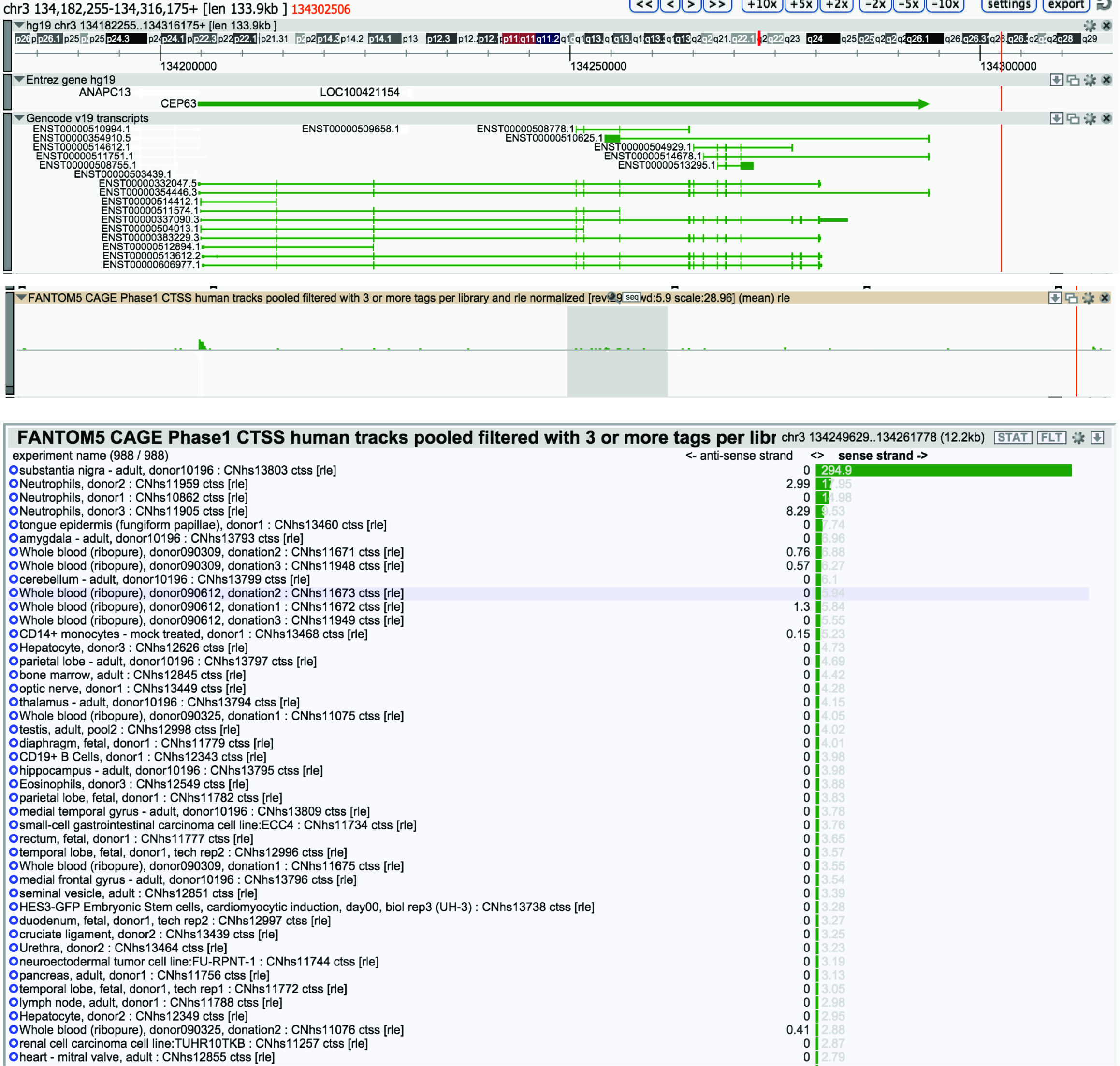

Supplement: Supplementary file 3 — Supplementary material 3: Fig. S3 FANTOM5 expression of neuronal TSS. The top panel shows the genomic context within which CEP63 (gene region marked in green) lies and the known transcripts below. The middle panel shows the levels of expression starting at each region. The green bars show expression of CEP63. The cluster of TSSes inside the CEP63 gene is highlighted (grey), and the bottom panel shows where the transcripts from this TSS cluster are expressed (green bars, sorted by expression levels). The highest expression is seen in the medial temporal gyrus (220.52 rle normalised tags/library) and only a few tissues show any expression > 10 rle normalised tags/library (an arbitrary cut-off for determining expression in a tissue). (TIFF 3256 kb) [file 439_2015_1602_MOESM3_ESM.tiff]

Extracellular Space

Plasma Membrane

Cytoplasm

Nucleus

Other

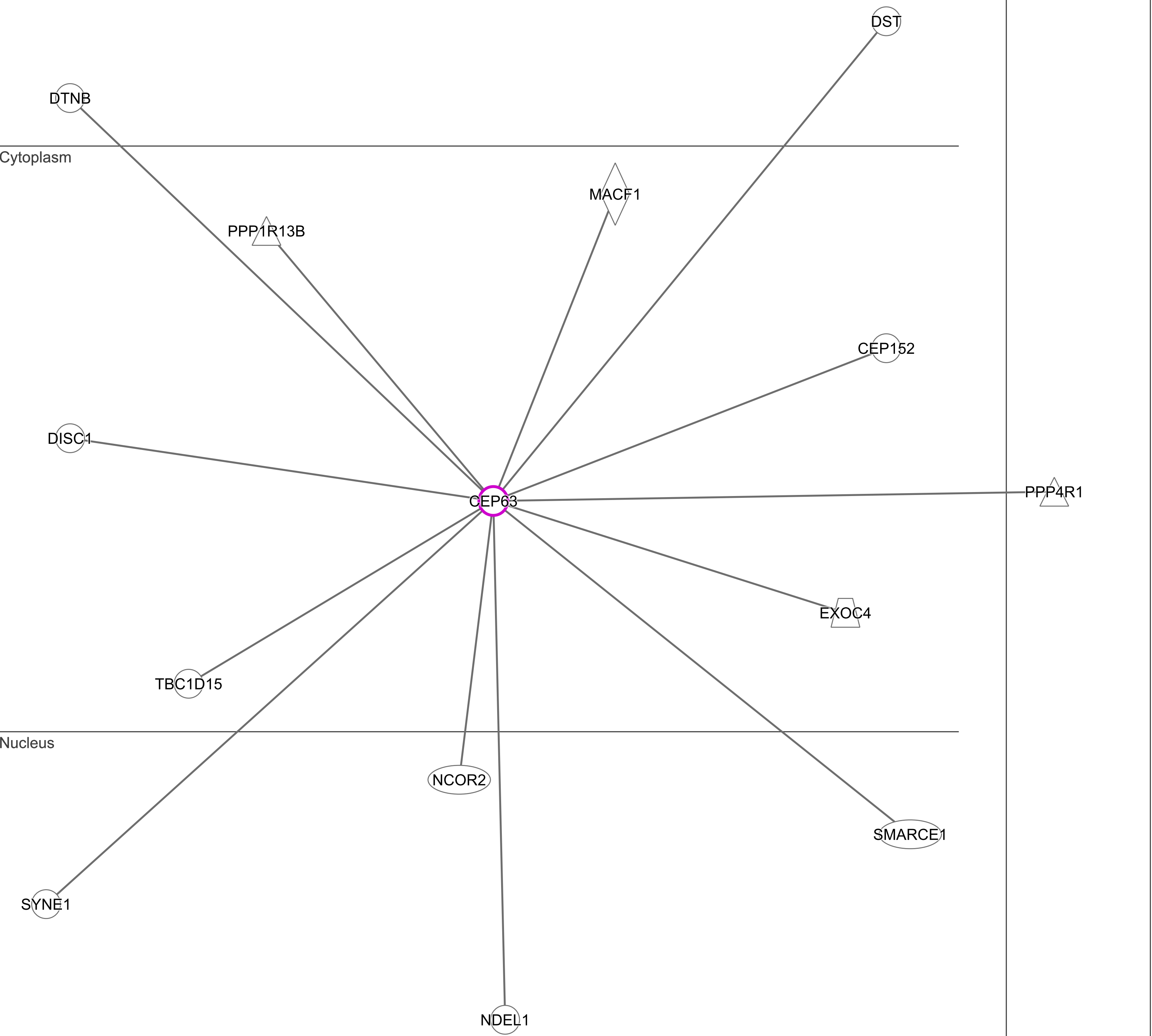

Supplement: Supplementary file 4 — Supplementary material 4: Fig. S4 Thirteen direct protein–protein binding partners for CEP63. All molecules were experimentally validated according to analysis criteria in the IPA software. The shapes of the individual proteins in the figure denote the different protein families that the binding partners belong to (e.g. protein phosphatases PPP4R1 and PPP1R13B). (PDF 864 kb) [file 439_2015_1602_MOESM4_ESM.pdf]

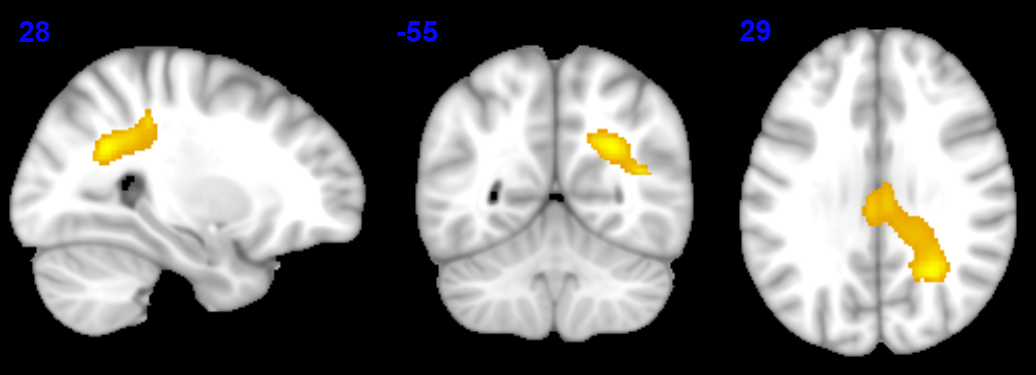

Supplement: Supplementary file 5 — Supplementary material 5: Fig. S5 Clusters (in yellow) from MRI data showing significant correlation between genotypes in rs7619451 and white matter volume in human brain. The AA/AC genotypes were significantly associated with larger white matter volume. The regions in the right hemisphere overlap with right superior longitudinal fasciculus and posterior part of corpus callosum (peak coordinate: 28, -55, 29; p = 0.0076, corrected at the cluster level with p < 0.01). (TIFF 1734 kb) [file 439_2015_1602_MOESM5_ESM.tif]
