# Supplementary material for: Mutation in CEP63 co-segregating with developmental dyslexia in a Swedish family
Source: Hum Genet. 2015 Sep 23;134:1239–48. doi: 10.1007/s00439-015-1602-1 (PMC4628622; doi:10.1007/s00439-015-1602-1)
Supplement: Supplementary file 8 — Supplementary material 8: Table S3 List of experimentally validated direct interacting protein partners to CEP63 based on Ingenuity analysis. (PDF 101 kb) [file 439_2015_1602_MOESM8_ESM.pdf]

Symbol	Ensembl Hs ID	Description
MACF1	ENSG00000127603	microtubule-actin crosslinking factor 1
EXOC4	ENSG00000131558	exocyst complex component 4
DTNB	ENSG00000138101	dystrobrevin, beta
SYNE1	ENSG00000131018	spectrin repeat containing, nuclear envelope 1
DISC1	ENSG00000162946	disrupted in schizophrenia 1
NCOR2	ENSG00000196498	nuclear receptor corepressor 2
SMARCE1	ENSG00000073584	SWI/SNF related, matrix associated, actin dependent regulator of chromatin, subfamily e, member 1
CEP152	ENSG00000103995	centrosomal protein 152kDa
DST	ENSG00000151914	dystonin
PPP1R13B	ENSG00000088808	protein phosphatase 1, regulatory (inhibitor) subunit 13B
TBC1D15	ENSG00000121749	TBC1 domain family, member 15
PPP4R1	ENSG00000154845	protein phosphatase 4, regulatory subunit 1
NDEL1	ENSG00000166579	nudE nuclear distribution gene E homolog (A. nidulans)-like 1
